# Agronomic Performance and Dual Resistance Evaluation to Powdery Mildew and Stripe Rust in 660 Wheat Germplasm Lines

**DOI:** 10.3390/plants15182859

**Published:** 2026-09-18

**Authors:** Kebei Chen, Pardeep Kumar, Guoyun Jia, Hao Chen, Yiduo Zhang, Hadi Bux, Shouhang Zheng, Xinli Zhou

**Affiliations:** 1 Wheat Research Institute, College of Life Sciences and Agri-Forestry, Southwest University of Science and Technology, Mianyang 621010, China; 2 National Key Laboratory of Green Pesticide, Key Laboratory of Green Pesticide and Agricultural Bioengineering, Ministry of Education, Center for R&D of Fine Chemicals, Guizhou University, Guiyang 550025, China; 3 Institute of Plant Sciences, University of Sindh, Jamshoro 76080, Pakistan; 4 Crop Germplasm Innovation and Genetic Improvement Key Laboratory of Sichuan Province, Mianyang Institute of Agricultural Science, Mianyang 621023, China

**Keywords:** wheat powdery mildew, stripe rust, resistance genes, wheat germplasm resources

## Abstract

Wheat powdery mildew (*Blumeria graminis* f. sp. *tritici*, *Bgt*) and stripe rust (*Puccinia striiformis* f. sp. *tritici*, *Pst*) are two foliar diseases that threaten global wheat production. The development of germplasms with resistance to both diseases and acceptable agronomic performance is a priority for improving wheat. A total of 660 wheat lines derived from crosses between elite commercial cultivars and resistant donors were evaluated for agronomic traits across five field environments (2019–2024), seedling resistance to *Bgt* races E15 and E17, and field resistance to stripe rust and were genotyped using markers linked to 14 known *Pm* loci. Resistance to *Bgt* was race dependent: only 22 lines (3.3%) exhibited high resistance to both E15 and E17, with infection types (ITs) ≤ 1 on the 0–4 powdery mildew scale, and resistance to the two races was weakly correlated, indicating race-specific variation. Stripe rust resistance was strongly environment dependent: the proportion of immune to highly resistant reactions (stripe rust IT ≤ 3 on the 0–9 scale) ranged from 94.4% (622/659) in 2020QL to 33.1% (218/659) in the highest-pressure environment (2022QL). At the line level, 382 of 659 lines (58.0%) had a mean stripe rust IT ≤ 3 across environments and were classified as immune or highly resistant. Marker analysis detected at least one marker allele linked to known *Pm* loci in 502 lines (76.1%), involving eight *Pm*-linked loci (*Pm1c*, *Pm2*, *Pm5e*, *Pm6*, *Pm30*, *Pm42*, *Pm45* and *Pm52*). Because these markers indicate the presence of linked or associated loci rather than direct functional validation, the detected loci are referred to here as marker-detected *Pm*-linked loci. Notably, resistance to *Bgt* was not simply proportional to the number of marker-detected *Pm*-linked loci. Lines carrying three marker-detected *Pm*-linked loci showed significantly lower infection types to E17 than lines carrying two or fewer loci (*p* < 0.05), whereas four-locus lines did not show additional resistance. This result suggests a possible combination-associated pattern rather than a strictly number-dependent relationship; however, this interpretation should be considered cautiously because marker-count and marker-combination classes differed in sample size. The MBH1 marker, which is linked to *Pm21* and *PmV*, was not detected in the overall group, suggesting that the identified resistant lines represent valuable resources without the MBH1-tagged *Pm21*/*PmV* background. Nineteen selected lines that combined high resistance to both stripe rust and powdery mildew with acceptable agronomic performance were identified. Among these, SWUST-675, SWUST-716, and SWUST-718 represent promising germplasms for disease-resistance breeding in wheat, particularly as lines with strong dual-disease resistance and without the MBH1-tagged *Pm21*/*PmV* background.

## 1. Introduction

Wheat (*Triticum aestivum* L.) is one of the most widely cultivated cereal crops and is a staple food for 40% of the global population. It provides more than 20% of human caloric intake and 25% of dietary protein. Wheat grain is a key source of carbohydrates and accounts for 55% of total carbohydrate intake from food [1]. Stable wheat production is therefore directly linked to global food security. However, wheat productivity is constrained by biotic stresses, including powdery mildew and stripe rust, two damaging foliar diseases. Powdery mildew is ranked among the eight most damaging pathogens for wheat worldwide [2], whereas stripe rust can cause severe epidemics and complete yield loss when susceptible cultivars are infected at early developmental stages [3]. Identifying and deploying effective resistance resources remains a priority in wheat improvement programs.

Wheat powdery mildew is caused by the obligate biotrophic fungus *Blumeria graminis* f. sp. *tritici* (*Bgt*), a specialized pathogen in the order Erysiphales. After infection, visible grayish-white powdery colonies consisting of conidiophores and conidia develop on leaf surfaces, reducing their photosynthetic capacity and causing chlorosis, premature senescence, impaired grain filling, and yield losses of 30–50% under severe epidemic conditions. Many powdery mildew resistance (*Pm*) genes have been discovered and characterized, with more than 140 *Pm* genes or alleles at 64 loci reported in wheat and its relatives [4,5], including 71 formally designated *Pm* genes (*Pm1*–*Pm71*). Many of these genes have been introgressed from wild relatives or related species, such as *Triticum urartu*, *Triticum durum*, and other Poaceae members, broadening the genetic basis of resistance in cultivated wheat.

Functional studies have clarified the roles of several *Pm* genes in powdery mildew resistance [6]. To date, 19 powdery mildew resistance genes have been cloned [7]. Most cloned *Pm* genes encode coiled-coil nucleotide-binding leucine-rich repeat (CNL) proteins in the NLR receptor family [8]. Examples include the *Pm3b*/*Pm8*/*Pm17* gene cluster [9], *Pm2a* [10], *Pm21*/*Pm12* [11,12], the *Pm60*/*MlIW172*/*MlWE18* cluster [13,14,15], *Pm5e* [16], *Pm55* [17], *Pm41* [18], *Pm1a* [19], *Pm69* [20], and *Pm6S1* [21]. Other cloned resistance genes belong to different functional classes: *Pm24* and *WTK4* encode tandem kinase proteins (TKPs) [22,23], whereas *Pm38*, corresponding to the *Lr34*/*Yr18*/*Sr57* gene cluster, encodes an ATP-binding cassette transporter [24,25], and *Pm46*, corresponding to *Lr67*/*Yr46*/*Sr55*, encodes a hexose transporter [26]. Recent work has identified additional resistance sources. *PmAeu1* from *Aegilops umbellulata* was cloned using BSR-Seq and PacBio sequencing, and the KASP marker XAeuNLR1 was developed for marker-assisted selection (MAS) [27]. The wild emmer-derived gene *PmLF540* on chromosome 4AL showed broad resistance and was validated using qRT–PCR and the KASP marker KASP540-1 [28]. The durum-derived *PmDR88* allele showed distinctive expression patterns and a broad resistance spectrum [29]. Genome-wide association analysis identified four stable QTLs, including two novel loci [30]. In addition, *PmCAHM* from a landrace was mapped to chromosome 1BS with linked SSR markers [31], and the *SuPm55*–*Pm55a* interaction was shown to regulate stage-specific resistance while balancing defense and yield-related effects [17].

Stripe rust, also known as yellow rust, is caused by *Puccinia striiformis* f. sp. *tritici* (*Pst*) and is a destructive fungal disease of wheat (*Triticum aestivum* L.), it is prevalent in key wheat-growing countries worldwide, including China, India, Pakistan, Australia, the United States, Mexico, and northwestern Europe, in temperate, medium-altitude, and maritime climates [32]. Yield losses can reach 100% under favorable epidemic conditions, with typical losses ranging from 10–70% [33]. In China, severe epidemics in 1950, 1964, 1990, and 2002 resulted in yield losses of 6.0, 3.2, 1.8, and 1.3 million tonnes, respectively. National surveys estimated that stripe rust affects approximately 4.2 million hectares of wheat each year in China, with severe epidemics causing yield losses of 10–70% [34]. Host resistance is considered the most economical and environmentally sustainable strategy for stripe rust management. Extensive efforts have been made to identify resistance sources, and more than 300 stripe rust resistance genes and QTLs have been identified across all 21 wheat chromosomes [35,36], many of which have recently been cloned and functionally defined. For example, the rye-derived stripe rust resistance gene *Yr9* was cloned from the 1BL.1RS translocation line Lumai15 using sequencing trait-associated mutation (STAM) technology. *Yr9* encodes a coiled-coil nucleotide-binding site leucine-rich repeat (CC-NBS-LRR) protein and belongs to a conserved NLR cluster orthologous to the barley *Mla* locus, which is located within the 1BL.1RS translocation region [37]. A global wheat core collection was developed, integrating 47,000 phenotypic datasets, and a map containing 431 *Yr*-QTLs was constructed. A total of 559 candidate genes (including *Yr5x* and *Yr6*/*Pm5*) and the confirmed gene *YrKB* (TaEDR2-B) were further identified, all of which are associated with resistance to multiple *Pst* races, resistance to two pathogen species, or broad-spectrum rust resistance without an apparent yield penalty [38]. These findings establish that the synergistic action of the *TdNLR1*/*TdNLR2* gene pair governs *YrTD121*/*Yr84* resistance in wild emmer wheat (*Triticum dicoccoides*), providing direct evidence for a functional NLR-mediated immune mechanism [39].

Powdery mildew and stripe rust both reduce wheat yield and are major breeding targets [40]. Current management relies mainly on fungicide application and resistant cultivars. Intensive fungicide use increases production costs, selects for fungicide-resistant pathogen populations, and may affect human health, beneficial organisms, and the environment [41,42,43]. Resistant cultivars provide a cost-effective and environmentally sustainable control strategy [44]. However, pathogen evolution driven by natural variation and environmental selection has reduced the effectiveness of many resistance genes. With respect to powdery mildew, several widely deployed genes, including *Pm1*, *Pm2*, *Pm3*, *Pm4*, *Pm5*, and *Pm8*, have lost or are rapidly losing effectiveness against prevalent *Bgt* isolates in several major wheat-producing regions of China [45,46], although the virulence structure of *Bgt* populations shows clear regional heterogeneity [47]. Loss of effectiveness is not always attributable to pathogen adaptation alone: in certain genetic backgrounds, a resistance gene can be suppressed by an interacting locus, as shown for the rye-derived *Pm8*, whose function is abolished by its wheat ortholog *Pm3* through formation of a heteromeric protein complex [48]. Even *Pm21*, derived from *Dasypyrum villosum* and historically regarded as highly effective, is facing an increasing risk of resistance breakdown due to strong selection pressure resulting from its large-scale, single-gene deployment in Chinese wheat production [49]. Similarly, several stripe rust resistance genes, including *Yr1*-*Yr4*, *Yr6*–*Yr10*, *Yr17*, *Yr20*–*Yr22*, *Yr24*–*Yr29*, and *Yr43*, have rapidly lost effectiveness in China because of race-specific variation in *Pst* populations [50].

In this study, we used molecular markers to determine the distribution of powdery mildew resistance loci among 660 wheat lines. We evaluated seedling-stage powdery mildew resistance and adult-plant stripe rust resistance under natural field conditions and characterized yield-related agronomic traits, including plant height (PH), spike length (SL), spikelet number per spike (SN), and thousand-grain weight (TGW), across field environments from 2019 to 2024 to assess their stability and interrelationships. By integrating marker screening, resistance phenotyping, and multiyear field trait assessment, we identified elite wheat lines that combine resistance to both diseases with desirable agronomic traits.

## 2. Materials and Methods

### 2.1. Plant Materials

The 660 wheat lines were developed by the Wheat Research Institute of Southwest University of Science and Technology. They were created by crossing currently recommended varieties (whose resistance to stripe rust has weakened or has been lost) with donor lines harboring stripe rust resistance genes (QTLs). This was followed by selection through molecular markers and field screening combined with backcrossing and multiple generations of selfing, resulting in extremely high resistance to the currently prevalent races of the stripe rust pathogen. These lines exhibited moderate to high resistance (infection types (ITs) of 2–4) to stripe rust under natural disease pressure in Sichuan (Mianyang) and Shanxi (Yangling) environments. There were a total of nine recurrent parents, originating from major wheat-producing regions such as Sichuan, Hebei, Henan, Hubei and Shandong. Previous studies have shown that Bainong Aikang 58 maintains high resistance to stripe rust, with ITs of 2–3; Zhengmai 9023, Handan 6172 and Chuanmai 42 have partially lost their resistance to stripe rust, with reaction types ranging from 4–6; and Lunxuan 987, Jimai 22, Xinmai 26, Xiangmai 25 and Yannong 21 no longer possess stripe rust resistance, with reaction types ranging from 7–9. Bainong Aikang 58 and Yannong 21 have partially lost their resistance to powdery mildew, with powdery mildew reaction types ranging from 2–3; Lunxuan 987 retains moderate resistance to powdery mildew, with a reaction type of 1–2 (Table 1), whereas the remaining six parental materials are susceptible to powdery mildew.

A total of 10 paternal accessions, all of which were spring wheat, were provided by Professor Chen Xianming of Washington State University, USA. PI 660057 carries the stripe rust resistance gene *Yr52* [51]; PI 660060 carries *Yr62* [52]; PI 660061 carries the gene *Yr59* [53]; PI 660076 carries the stripe rust resistance QTLs *QYr076.jaas-2A*, *QYr076.jaas-4D.1*, and *QYr076.jaas-4D.2* [54]. PI 660115 carries an unknown stripe rust resistance gene; field trials indicate that it is highly resistant to stripe rust [55]; PI 660122 carries the QTL *QYrPI660122.swust-4DS* [55]; PI 610750 carries *Yr48* [56]; AvS/Exp F_7_ carries *YrExp2* [57]; and AvS/Alp F_7_-71 carries *Yr39* [58]; and the wheat line P9897 carries two major-effect QTLs for stripe rust resistance [59], *QYr.nafu-2BL* and *QYr.nafu-3BS* [60] (Table 2). In the preliminary phase of this study, 29 hybrid combinations were constructed, and following backcrossing to high generations combined with field screening, a total of 660 wheat lines were obtained (Table S1).

### 2.2. Molecular Markers

Fourteen pairs of molecular markers linked to 14 known powdery mildew resistance genes (*Pm1c*, *Pm2*, *Pm4a*, *Pm5e*, *Pm6*, *Pm21*/*PmV*, *Pm24*, *Pm30*, *Pm33*, *Pm35*, *Pm42*, *Pm45*, *Pm52* and *Pm68*) (Table 3) were used to genotype the 660 wheat lines. *Pm21* and *PmV* were detected with the same MBH1 marker because the two loci share this marker; they are therefore treated as a single marker-detected unit (*Pm21*/*PmV)* rather than two independent loci. It should be noted that the markers used in this study were developed based on reported linkage relationships with *Pm* loci. Therefore, marker detection indicates the presence of associated genomic regions but does not directly confirm the presence, integrity, or expression of functional resistance genes. Allelic variation, recombination between marker loci and resistance genes, and genetic background effects may influence the relationship between marker detection and resistance phenotype. All primer information and associated experimental protocols were provided by the Yantai Academy of Agricultural Sciences (Shandong Province).

### 2.3. Greenhouse Assay

The wheat lines were sown in 128-well plug trays (2 × 2 cm per cell) and grown under controlled greenhouse conditions at the Wheat Research Institute, Southwest University of Science and Technology, Mianyang, China. The photoperiod and temperature regime were set to 18 h light at 20 °C and 6 h darkness at 18 °C, with the relative humidity maintained between 50 and 70%. Before inoculation, sterile water was sprayed around the trays to limit the dispersal of *Bgt* spores. For inoculation, infected leaves collected from susceptible plants were gently shaken over the test seedlings to uniformly distribute *Bgt* spores onto the leaves. After inoculation, the trays were covered with transparent plastic lids, and the ventilation ports were sealed with gauze to prevent spore escape and cross-contamination. Disease symptoms were evaluated approximately 14 days after inoculation using an IT scale of 0–4 [72]: 0 = immune (no visible lesions or very small necrotic flecks, no spores or hyphae), 1 = highly resistant (small lesions with chlorosis and necrosis and little or no sporulation), 2 = moderately resistant (larger necrotic lesions with moderate chlorosis and sparse sporulation), 3 = moderately susceptible (visible sporulation covering 20–40% of the leaf area), and 4 = highly susceptible (dense sporulation covering most of the leaf surface). To avoid ambiguity, powdery mildew infection types scored on this 0–4 scale are abbreviated as ITpm hereafter, whereas stripe rust infection types scored on the separate 0–9 scale (Section 2.4) are abbreviated as ITsr.

### 2.4. Field Experiments

Field experiments were conducted across five environments, designated 2019QL, 2020QL, 2020YL, 2022QL, and 2024XJ, to evaluate agronomic performance and stripe rust resistance under natural field disease conditions. The 2019QL, 2020QL, and 2022QL trials were conducted during the 2018–2019, 2019–2020, and 2021–2022 growing seasons, respectively, at Qinglian (QL) Experimental Farm, Mianyang, Sichuan Province, China (31°40′ N, 104°39′ E). The 2020YL trial was conducted during the 2019–2020 growing season at Caoxinzhuang Experimental Farm of Northwest Agriculture and Forestry University, Yangling (YL), Shanxi Province, China (34°20′ N, 108°07′ E). The 2024XJ trial was conducted during the 2023–2024 growing season at the Xiaojian (XJ) Experimental Station of Mianyang Academy of Agricultural Sciences, Mianyang, Sichuan Province, China (31°46′ N, 104°39′ E). Each wheat line was sown in a single 1-m row with 30 cm between rows, and 20–30 seeds were evenly distributed in each row. Four agronomic traits, namely plant height (PH), spike length (SL), spikelet number per spike (SN), and thousand-grain weight (TGW), were recorded after the plants reached the milk stage to ensure consistent phenotypic assessment. Plant height was measured from the soil surface to the spike tip, excluding awns. At maturity, the main-spike length was measured from the first fertile spikelet at the base to the spike tip using three representative spikes per row, and the number of spikelets was counted on the same spikes. For each trait, measurements from three randomly selected plants per row were averaged. After physiological maturity, the grains were harvested, threshed, and thoroughly dried, and the thousand-grain weight was determined by weighing three samples of 1000 grains per line. Stripe rust was evaluated for each row from heading (Z50) to grain filling (Z80) [73] under natural field disease conditions.

Field disease responses were evaluated at the stage when stripe rust was fully developed on the susceptible check Mingxian 169 (MX169), i.e., when disease severity on the check had reached the level at which differences among genotypes were most clearly expressed in each environment. That disease pressure differed substantially among environments is evident at the population level: mean disease severity across the 660 lines ranged from 9.7% in 2020QL to 28.1% in 2022QL, and the proportion of observations with severity above 20% increased from 7.9% to 69.2%. The stripe rust infection type (ITsr) and disease severity (DS) were recorded twice for the 18 parental lines and their derived selected lines. ITsr was scored using a 0–9 scale [74], where 0 = immune, 1–3 = highly resistant, 4–6 = moderately resistant, and 7–9 = susceptible. DS was recorded as the percentage of leaf area affected by stripe rust under natural field disease conditions using the following scale: 0, 5, 10, 15, 20, 25, 30, 35, 40, 45, 50, 55, 60, 65, 70, 75, 80, 85, 90, 95, or 100. The two observations were averaged for subsequent analysis.

### 2.5. DNA Extraction and PCR

Fresh leaf samples (2–3 cm) were collected from field-grown plants, ground in liquid nitrogen using a Geno/Grinder 2010 tissue grinder (SPEX SamplePrep, Metuchen, NJ, USA), and genomic DNA was extracted using a modified cetyltrimethylammonium bromide (CTAB) method [75]. DNA was extracted from both parental lines and BC_1_F_4:5_ lines for genotyping, and DNA quality and quantity were determined using a NanoDrop ND-1000 spectrophotometer (Thermo Scientific, Wilmington, NC, USA). PCR amplification was performed in a 10 μL reaction volume containing 1 μL of 10× Taq buffer, 0.8 μL of 10 mM dNTPs, 0.2 μL of Taq DNA polymerase (5 U/μL), 1 μL each of forward and reverse primers (10 μM each), 2 μL of genomic DNA (200 ng/μL), and 4 μL of double-distilled water (ddH_2_O). The PCR cycling program consisted of initial denaturation at 95 °C for 5 min; 35 cycles of denaturation at 94 °C for 30 s, annealing at the primer-specific temperature for 30 s, and extension at 72 °C for 30 s; and a final extension at 72 °C for 8 min. PCR products were separated via 6% polyacrylamide gel electrophoresis (PAGE).

### 2.6. Statistical Analysis

All statistical analyses were performed using Python 3.9 (Python Software Foundation, Wilmington, DE, USA) with the pandas (https://pandas.pydata.org, accessed on 20 June 2026), NumPy (https://numpy.org, accessed on 20 June 2026), SciPy (https://scipy.org, accessed on 20 June 2026), statsmodels (https://www.statsmodels.org, accessed on 20 June 2026) [76], and matplotlib (https://matplotlib.org, accessed on 20 June 2026) [77] packages. Descriptive statistics, including the minimum, maximum, mean, standard deviation, coefficient of variation (CV), skewness, and kurtosis, were calculated for agronomic traits and disease-resistance traits in each environment. The coefficient of variation was calculated as CV (%) = standard deviation/mean × 100.

Pearson correlation coefficients were calculated to evaluate relationships among agronomic traits, stripe rust infection type (IT), stripe rust disease severity (DS), and powdery mildew infection types to *Bgt* races E15 and E17. Differences in powdery mildew infection types between *Bgt* races E15 and E17 were assessed using the Mann–Whitney U test because infection-type scores are ordinal disease-response data.

Two-way analysis of variance (ANOVA) was performed using statsmodels to evaluate the effects of genotype, environment, and genotype-by-environment interaction on agronomic traits and stripe rust resistance traits. Best linear unbiased prediction (BLUP) values and variance components were estimated using mixed linear models in statsmodels. Broad-sense heritability (H^2^) was estimated on an entry-mean basis using variance components derived from the mixed model. Figures were generated using matplotlib. Statistical significance was defined as *p* < 0.05, *p* < 0.01, and *p* < 0.001. For marker-detected *Pm*-linked locus combinations, descriptive comparisons were performed because several combination classes contained limited numbers of lines. Therefore, differences among rare combinations were interpreted cautiously rather than as definitive evidence of combination-specific resistance effects.

Because the trials were unreplicated within environments, the genotype × environment interaction mean square was used as the error term for testing the genotype and environment main effects. Consequently, the G × E interaction could not be tested for significance, and its magnitude was assessed descriptively using variance components estimated from the expected mean squares.

The stability of stripe rust resistance across environments was assessed descriptively rather than by formal significance testing. For each line, the number of environments in which the infection type met the resistance threshold (IT ≤ 3) was counted, and the proportion of lines achieving this threshold in all four environments was compared between the 19 selected elite lines and the full population using Fisher’s exact test. Performance in the highest-pressure environment (2022QL) was additionally examined as an independent indicator of stability under severe disease pressure.

### 2.7. Selection Criteria for Elite Lines

Elite lines were selected by integrating disease-resistance performance and agronomic traits. For disease resistance, lines were required to show high resistance or immunity to both *Bgt* races E15 and E17 (IT ≤ 1) and high resistance to stripe rust under field conditions (*Pst* IT ≤ 3 and DS ≤ 20%). For agronomic performance, lines were required to maintain acceptable or desirable values for major yield-related traits: PH, 60–115 cm; TGW, ≥40 g; SN, ≥14; SL, ≥8 cm. Lines meeting both the disease-resistance and agronomic-performance criteria were considered elite germplasm lines.

## 3. Results

### 3.1. Agronomic Variation Across Environments

**Figure 1:**
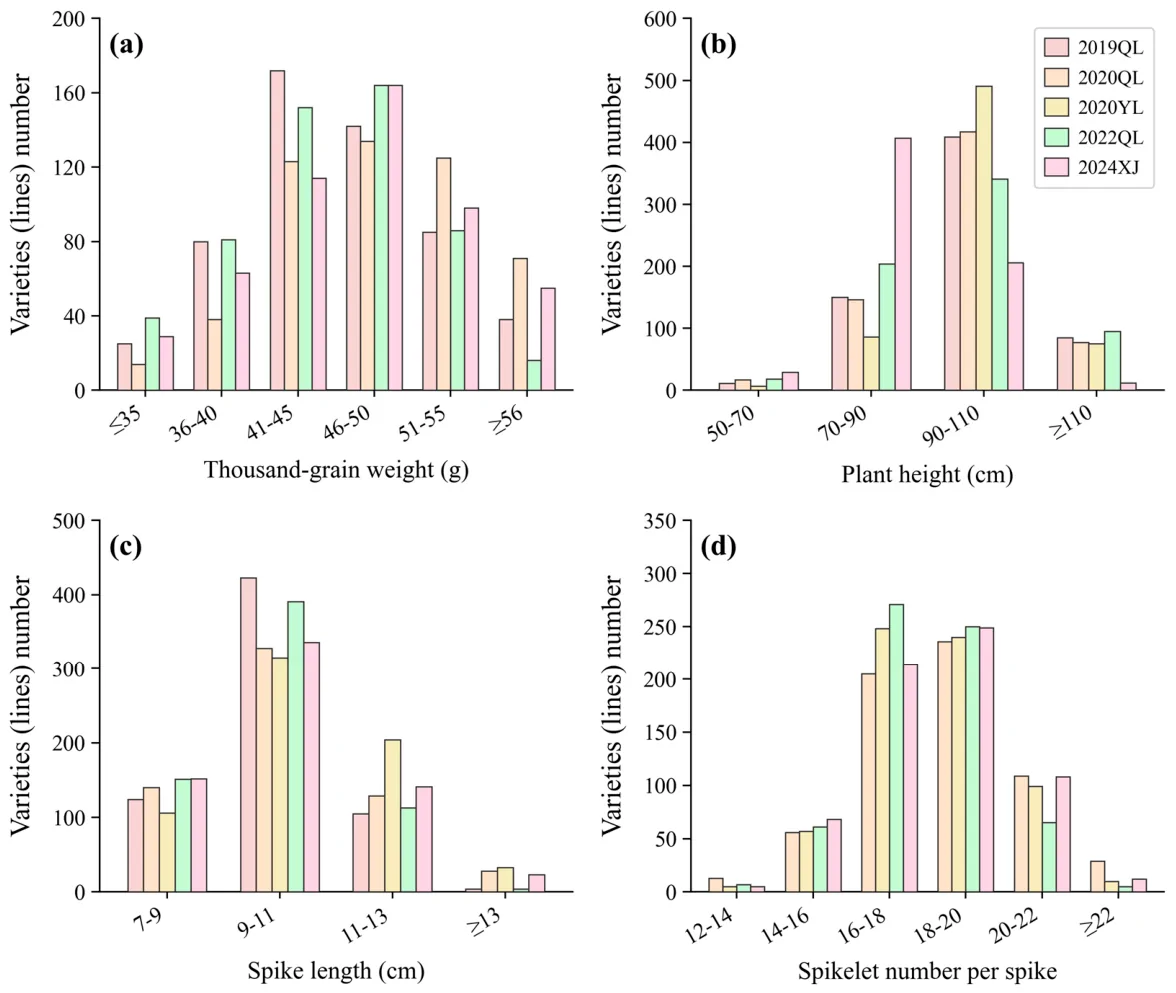
Distribution of agronomic traits in the 660 wheat lines across five environments. (**a**) Thousand-grain weight (TGW). (**b**) Plant height (PH). (**c**) Spike length (SL). (**d**) Number of spikelets per spike (SN). Different colors represent 2019QL, 2020QL, 2020YL, 2022QL, and 2024XJ.

Across the five field environments, the 660 wheat lines showed broad continuous variation in major agronomic traits, while most observations remained within the practical breeding range (Figure 1). With respect to thousand-grain weight (TGW), 27.7% and 28.3% of the valid observations were distributed in the 41–45 g and 46–50 g classes, respectively; together, 56.0% of the observations were within the 41–50 g range. Observations below 35 g and above 56 g accounted for 4.1% and 7.5%, respectively (Figure 1a). With respect to plant height (PH), 60.7%, 63.3%, 71.9%, and 51.7% of the lines were in the 90–110 cm class in 2019QL, 2020QL, 2020YL, and 2022QL, respectively. In 2024XJ, the distribution shifted toward shorter plants, with 61.9% of the lines in the 70–90 cm class (Figure 1b). With respect to the spike length (SL), 52.6% of the valid observations were in the 9–11 cm class, followed by 20.9% in the 11–13 cm class. Only 1.6% of the observations were shorter than 7 cm, and 1.9% were longer than 13 cm (Figure 1c). With respect to spikelet number per spike (SN), 34.3% and 36.8% of the valid observations were in the 16–18 and 18–20 classes, respectively; 71.2% of the observations were between 16 and 20 spikelets per spike. Only 1.2% of the observations exceeded 22 spikelets per spike (Figure 1d). Therefore, the population retained phenotypic diversity while maintaining agronomically acceptable plant architecture.

### 3.2. Correlations Among Agronomic Traits

**Figure 2:**
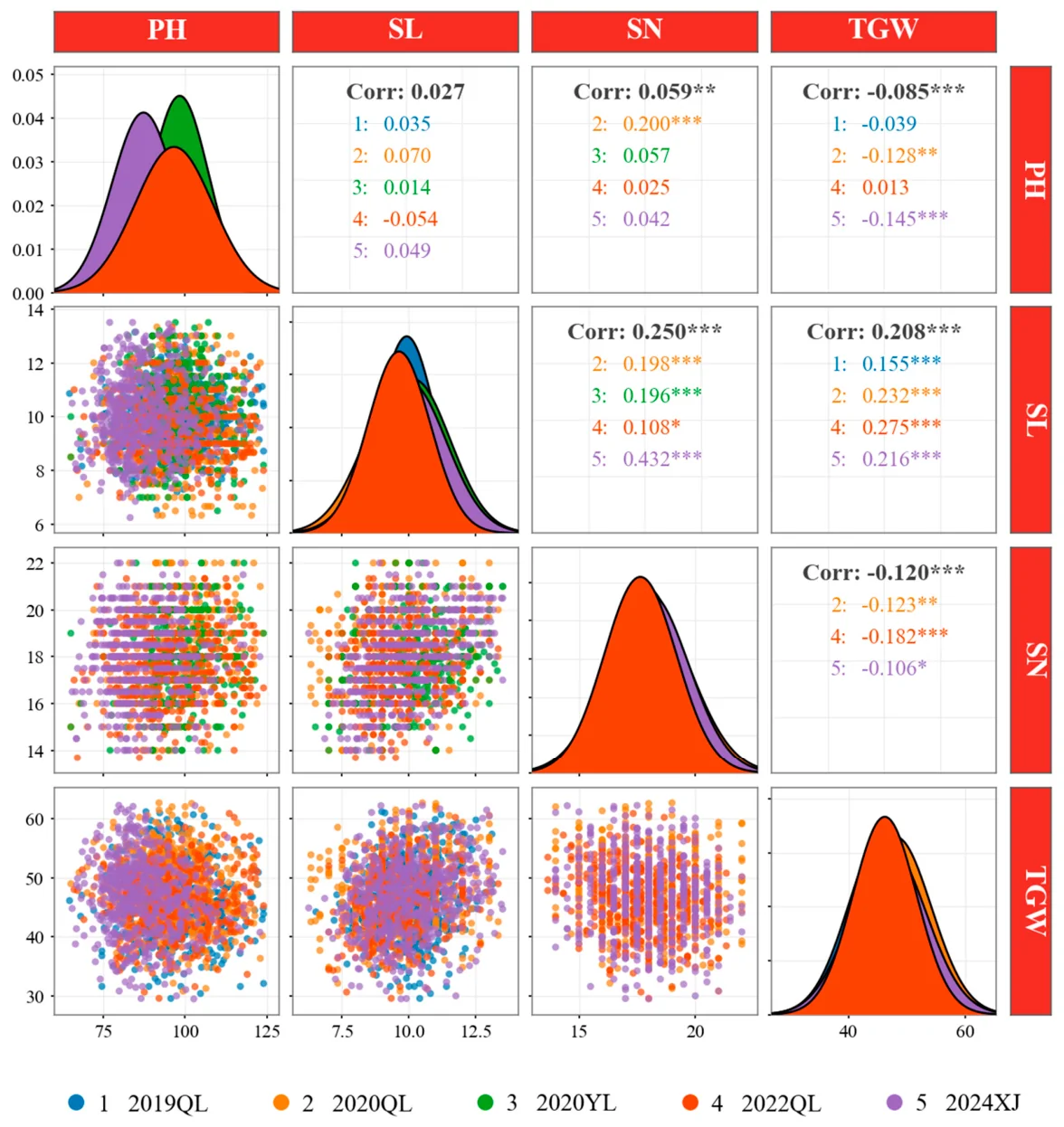
Pairwise relationships among the agronomic traits in the 660 wheat lines. The diagonal panels show density distributions of plant height (PH), spike length (SL), number of spikelets per spike (SN), and thousand-grain weight (TGW); the (**lower**) panels show scatter plots; the (**upper**) panels show Pearson correlation coefficients across environments. Numbers 1–5 correspond to 2019QL, 2020QL, 2020YL, 2022QL, and 2024XJ, respectively. Asterisks indicate significant correlations: * *p* < 0.05, ** *p* < 0.01, *** *p* < 0.001; values without asterisks are not significant (*p* ≥ 0.05).

Pairwise correlation analysis revealed that the four agronomic traits differed in terms of the strength and consistency of their associations across environments (Figure 2). PH showed negligible associations with SL (r = 0.027), SN (r = 0.059), and TGW (r = −0.085). Although statistically significant (*p* < 0.05), the correlation coefficients for PH with SN and TGW were less than 0.1, indicating that plant height was largely independent of spike- and grain-related traits in this population. SL showed stronger positive associations with SN (r = 0.250) and TGW (r = 0.208). The SL–SN association was positive across the environments where it was detected, with the strongest correlation in 2024XJ (r = 0.432), followed by 2020QL (r = 0.198), 2020YL (r = 0.196), and 2022QL (r = 0.108). Similarly, positive associations between SL and TGW were observed for 2019QL (r = 0.155), 2020QL (r = 0.232), 2022QL (r = 0.275), and 2024XJ (r = 0.216). SN showed a weak negative association with TGW at the overall level (r = −0.120), with significant negative correlations in 2020QL (r = −0.123), 2022QL (r = −0.182), and 2024XJ (r = −0.106). SL was positively correlated with SN (r = 0.250) and TGW (r = 0.208). SN was weakly negatively correlated with TGW (r = −0.120).

### 3.3. Seedling Responses to *Bgt* Races E15 and E17

**Figure 3:**
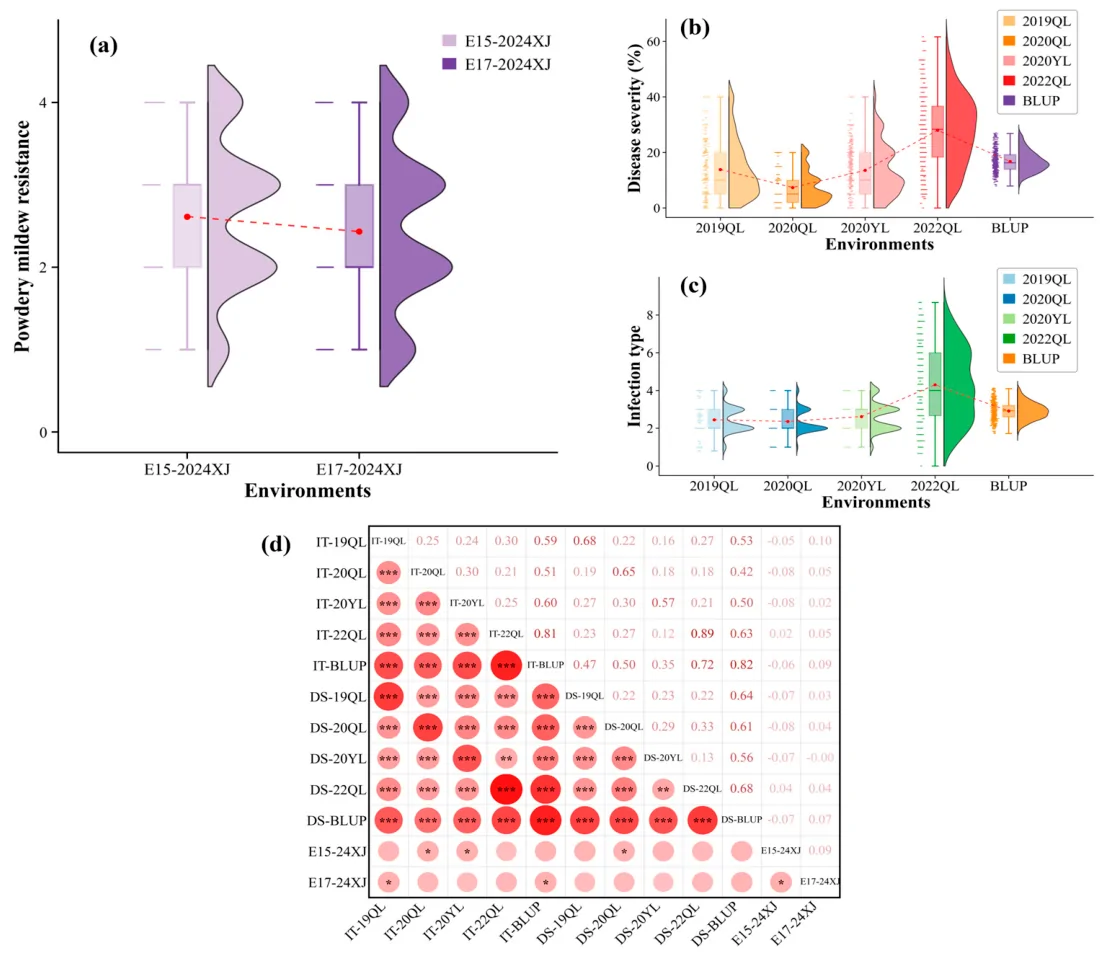
(**a**) Seedling infection-type distribution after inoculation with *Bgt* races E15 and E17 in 2024XJ. Disease resistance performance and correlation analysis of the 660 wheat lines. (**b**) Stripe rust disease severity distributions across the 2019QL, 2020QL, 2020YL, 2022QL, and BLUP estimates. (**c**) Stripe rust infection type distributions across 2019QL, 2020QL, 2020YL, 2022QL, and BLUP estimates. (**d**) Correlation matrix among stripe rust infection type (IT), stripe rust disease severity (DS), and powdery mildew infection type to E15 and E17. Asterisks indicate significant correlations: * *p* < 0.05, ** *p* < 0.01, *** *p* < 0.001. In (**c**), the red dotted lines denote the fitted normal distribution curves. In (**d**), the size and color intensity of the circles are proportional to the absolute value of the correlation coefficient, with larger and darker circles indicating stronger correlations.

**Figure 4:**
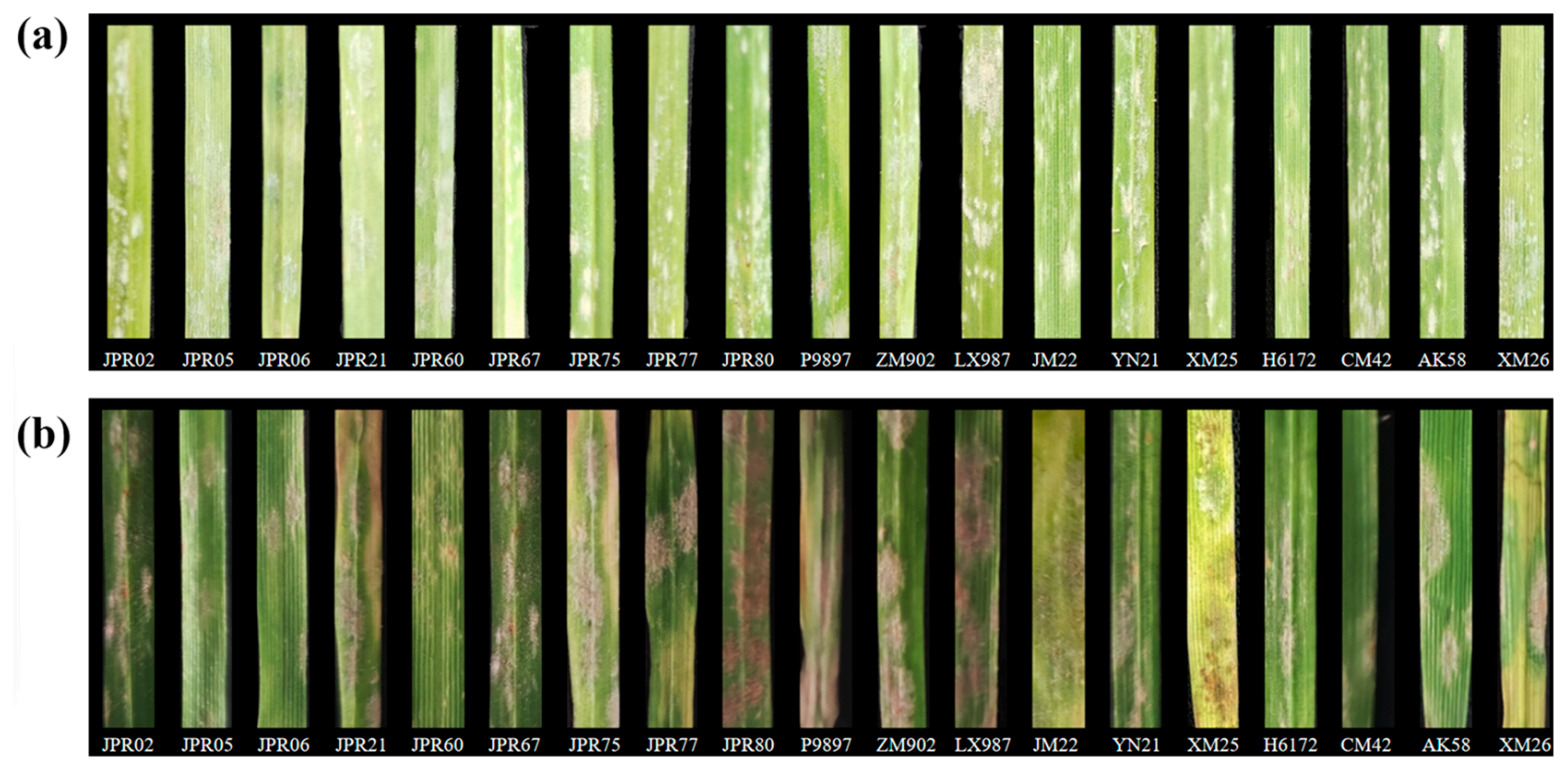
Phenotypic responses of parental wheat lines to powdery mildew after inoculation with *Bgt* races E15 and E17. The upper (**a**) and lower (**b**) rows show representative leaf reactions of the tested parental lines inoculated with races E15 and E17, respectively.

Seedling-stage responses to the two *Bgt* races, E15 and E17, differed among the tested wheat lines (Figure 3a). The infection types for both races ranged from resistant to susceptible, indicating that powdery mildew resistance was present in the population but differed across the *Bgt* races. Representative phenotypic responses of the parental wheat lines further illustrated variation in infection type among genotypes and between the two races (Figure 4). The mean infection type was lower for E17 (2.43) than for E15 (2.61), and the difference between the two races was significant (U = 227,305.00, *p* = 0.000530), indicating slightly stronger seedling resistance to E17 than to E15. Only 22 lines (3.3%) were highly resistant to both races (IT ≤ 1 to both E15 and E17). Thus, only a small subset of the population had high-level resistance to both *Bgt* races, indicating clear race-specific variation. Resistance to one *Bgt* race should not be assumed to represent broad-spectrum resistance to other *Bgt* races.

### 3.4. Stripe Rust Resistance Under Natural Field Disease Conditions

Stripe rust resistance under natural field disease conditions showed stronger environmental differentiation than did the seedling powdery mildew response (Figure 3b–d). The evaluation timing was determined based on disease development in the susceptible check MX169, which had reached the level of disease development at which genotypic differences were most clearly expressed, ensuring sufficient disease pressure. Consistent with this, population-level disease pressure was highest in 2022QL, where mean disease severity reached 28.1% and 69.2% of observations exceeded 20% severity, compared with 9.7% and 7.9%, respectively, in 2020QL for reliable discrimination among genotypes. Stripe rust infection type (IT) values were concentrated mainly in the low to moderate range in 2019QL, 2020QL, and 2020YL, whereas the 2022QL distribution shifted upward and became broader, indicating greater disease pressure and better discrimination among lines in that environment (Figure 3c). Disease severity (DS) showed a similar pattern: the lowest average disease level occurred in 2020QL, whereas 2022QL had the highest severity and the widest distribution (Figure 3b). Best linear unbiased prediction (BLUP) integrates multiple environmental responses and produces intermediate IT and DS distributions. Correlation analysis revealed that IT and DS were consistently and positively associated, with the strongest correlation in 2022QL (r = 0.89) and high correlations between the BLUP values for IT and DS (r = 0.82) (Figure 3d). The correlations between powdery mildew infection types and stripe rust traits were weak, indicating that resistance to *Bgt* and *Pst* was largely independent in this population.

The distribution of valid stripe rust scores also showed environmental differentiation. For IT, the numbers of valid observations were 659 in each of the four environments (2019QL, 2020QL, 2020YL and 2022QL). Immune or highly resistant reactions (IT ≤ 3) accounted for 86.2% (568/659), 94.4% (622/659), and 85.1% (561/659) of the valid observations in 2019QL, 2020QL, and 2020YL, respectively, but decreased to 33.1% (218/659) in 2022QL. In contrast, moderately resistant reactions (3 < IT ≤ 6) increased to 46.7% (308/659), and susceptible reactions (IT > 6) reached 20.2% (133/659) in 2022QL. DS followed the same trend: the proportions of observations with DS > 20% were 25.9% (171/659), 7.9% (52/659), and 19.4% (128/659) in 2019QL, 2020QL, and 2020YL, respectively, but increased to 69.2% (456/659) in 2022QL. The correlations between IT and DS were positive across environments (r = 0.821, 0.673, 0.775, and 0.882 for 2019QL, 2020QL, 2020YL, and 2022QL, respectively), indicating that the two indices captured similar variation in the stripe rust response. To summarize stripe rust resistance at the line level, the mean IT of each line across all environments with valid data was calculated; on this basis, 382 of 659 lines (58.0%) had a mean IT ≤ 3 and were classified as immune or highly resistant. This line-level proportion is markedly lower than most single-environment values because the pronounced upward shift of IT in the high-pressure 2022QL environment raised the multi-environment mean of many lines above the IT ≤ 3 threshold.

### 3.5. Two-Way ANOVA of Agronomic and Disease-Resistance Traits

Two-way ANOVA revealed that both genotype and environment significantly affected the six evaluated traits (PH, SL, SN, TGW, IT, and DS; Table 4). Genotype effects were highly significant for PH, SL, SN, TGW, IT, and DS, indicating genetic variation for both agronomic performance and disease response among the 660 lines. The environmental effects were also significant for all the traits and were strongest for disease traits, with much larger F values for IT (538.34 ***) and DS (646.42 ***) than for most agronomic traits. Broad-sense heritability was higher for agronomic traits, especially PH (79.42%) and TGW (70.14%), than for disease traits, whereas IT (46.69%) and DS (52.30%) showed moderate heritability. Agronomic traits were relatively stable but still environmentally sensitive, whereas the field stripe rust response depended strongly on the testing environment.

Descriptive statistics by environment supported these patterns (Table 5). The mean PHs were similar across 2019QL, 2020QL, 2020YL, and 2022QL (95.43–98.28 cm) but decreased to 86.43 cm in 2024XJ, consistent with the shift toward shorter plant height classes shown in Figure 1b. The mean SL ranged from 9.67–10.23 cm, and the mean SN remained stable at 17.79–18.02 spikelets per spike across the environments. TGW varied within a moderate range of 45.22–48.28 g, with the highest mean in 2020QL and the lowest in 2022QL. TGW and other yield-related traits showed continuous variation across environments.

Disease resistance traits were more environmentally dependent than were agronomic traits. Stripe rust IT and DS were lowest in 2020QL (mean IT = 2.36; mean DS = 7.36%) and highest in 2022QL (mean IT = 4.30; mean DS = 27.99%), supporting the interpretation that 2022QL had the strongest disease pressure and the greatest degree of phenotypic discrimination. Comparisons between *Bgt* races also revealed a significant difference between E15 and E17, with E15 causing a higher mean infection type than E17. Together with the moderate heritability of IT and DS, these results support the use of multienvironment field evaluation and multirace seedling screening when selecting wheat germplasms with stable disease resistance. The moderate heritability of disease traits and significant environmental effects indicate that resistance evaluation should integrate multi-environment testing rather than relying on single-environment performance.

### 3.6. Detection and Distribution of Marker Loci Linked to Pm Genes

**Figure 5:**
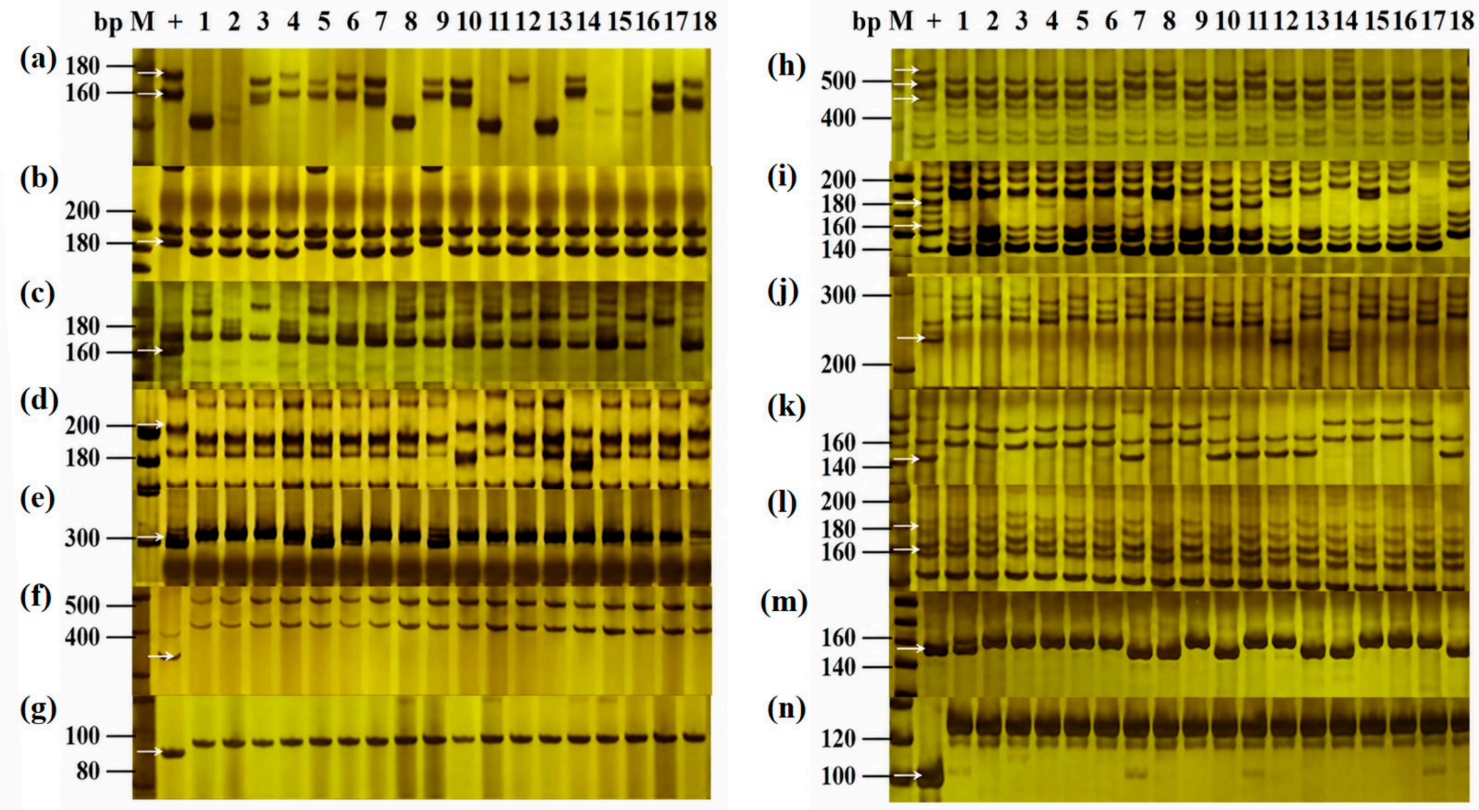
Electrophoretic detection of PCR products for markers linked to known powdery mildew resistance genes in parental lines. M represents the DNA ladder, + represents the positive control, and lanes 1–18, from (**left**) to (**right**), represent the tested parental materials listed in Table 6. White arrows indicate marker bands associated with resistant alleles. The marker panels correspond to the loci used for detecting the target *Pm* genes listed in Table 3. (**a**) Specific marker Xbarc78-F (for *Pm1c*), (**b**) specific marker XCFD81 (for *Pm2*), (**c**) specific marker Xgwm356 (for *Pm4a*), (**d**) specific marker WMC364 (for *Pm5e*), (**e**) specific marker CIT02g-18 (for *Pm6*), (**f**) specific marker MBH1 (for *Pm21*/*PmV*), (**g**) specific marker STS-*Pm24* (for *Pm24*), (**h**) specific marker Xgwm159 (for *Pm30*), (**i**) specific marker GWM111 (for *Pm33*), (**j**) specific marker CFD26 (for *Pm35*), (**k**) specific marker Xgwm148 (for *Pm42*), (**l**) specific marker XCFD80 (for *Pm45*), (**m**) specific marker Xicscl795 (for *Pm52*), and (**n**) specific marker Xdw15 (for *Pm68*).

**Figure 6:**
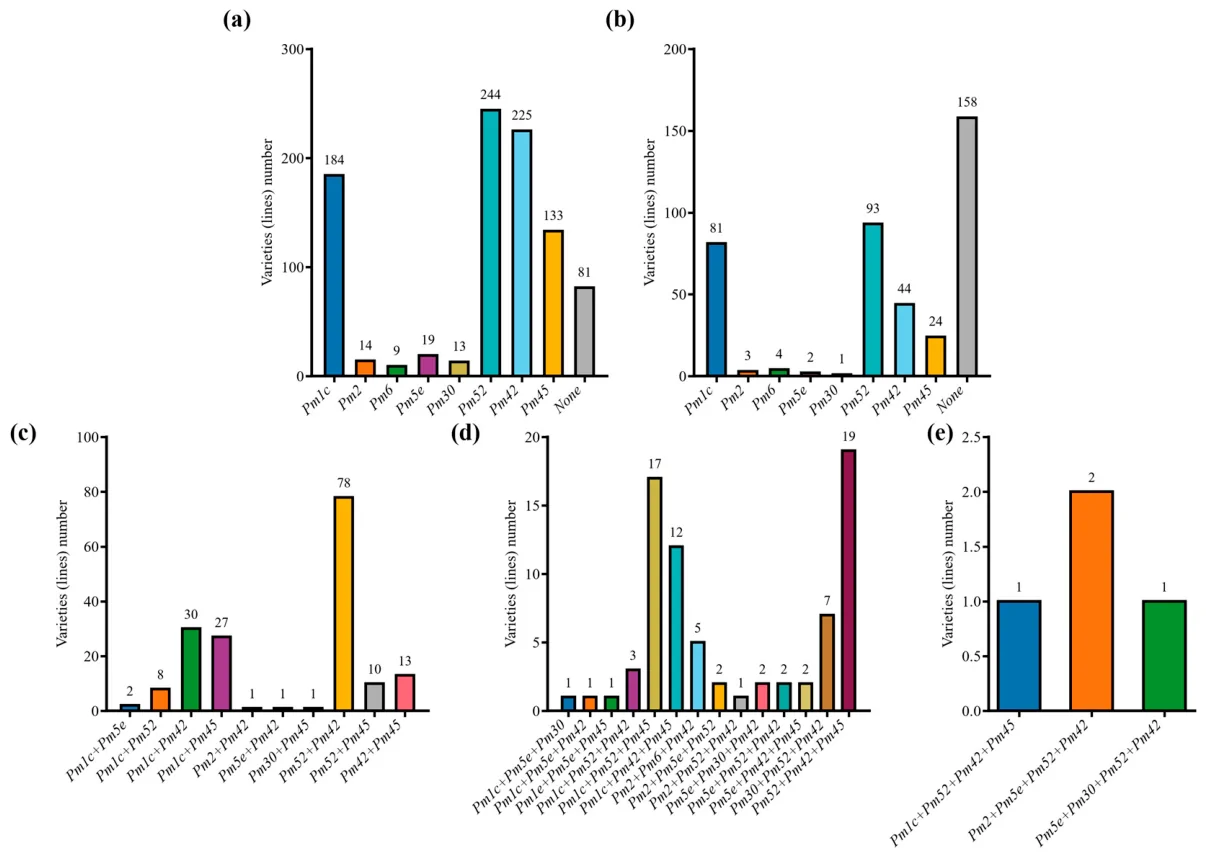
Distribution of marker-detected *Pm*-linked loci and marker-detected *Pm*-linked locus combinations in the 660 wheat lines. (**a**) Frequencies of individually marker-detected *Pm*-linked loci and lines without the tested markers. (**b**) Distribution of single-gene classes. (**c**) Distribution of two-gene combinations. (**d**) Distribution of three-gene combinations. (**e**) Distribution of four-gene combinations.

Molecular marker analysis detected marker alleles linked to several known powdery mildew resistance loci in the parental lines and derived population. (Figure 5 and Figure 6; Table 6). Among the 14 target genes, *Pm1c*, *Pm2*, *Pm5e*, *Pm6*, *Pm30*, *Pm42*, *Pm45*, and *Pm52* were detected in the parental materials, whereas *Pm4a*, *Pm21*/*PmV*, *Pm24*, *Pm33*, *Pm35*, and *Pm68* were not detected. *Pm21* and *PmV* were assayed with the same MBH1 marker because the two loci share this marker; therefore, they are treated as a single marker-detected unit (*Pm21*/*PmV*) rather than two independent loci. Among the 660 wheat lines, *Pm52* and *Pm42* were the most frequently detected genes, occurring in 244 and 225 lines, respectively, followed by *Pm1c* (184 lines) and *Pm45* (133 lines). *Pm2*, *Pm5e*, *Pm6*, and *Pm30* occurred at much lower frequencies. The MBH1 marker (associated with both *Pm21* and *PmV*) was not detected, indicating that no MBH1-tagged *Pm21*/*PmV* haplotype was detected in this population. Since MBH1 is a linked marker rather than a diagnostic functional marker, the absence of MBH1 amplification cannot completely exclude the presence of functional *Pm21*-related alleles. The individual marker distribution revealed that the population was enriched for a limited set of *Pm* loci rather than a broad, balanced set of the tested genes.

Gene combination analysis revealed that single genes and no marker classes represented the largest proportion of the population (Figure 6b). The main single-gene classes were *Pm52* alone (93 lines), *Pm1c* alone (81 lines), *Pm42* alone (44 lines), and *Pm45* alone (24 lines), whereas *Pm* markers were not detected in 158 lines in the combination analysis. Two gene combinations were also common (Figure 6c), especially *Pm52* + *Pm42* (78 lines), *Pm1c* + *Pm52* (30 lines), and *Pm1c* + *Pm45* (27 lines). Three gene combinations occurred in 69 lines, and four lines carried four gene combinations (Figure 6d,e). According to the line-level marker and phenotypic data, the four-gene class consisted of SWUST-224 and SWUST-237 (*Pm2* + *Pm5e* + *Pm52* + *Pm42*), SWUST-242 (*Pm5e* + *Pm30* + *Pm52* + *Pm42*), and SWUST-537 (*Pm1c* + *Pm52* + *Pm42* + *Pm45*). The number of lines and the corresponding *Bgt* responses for each marker-detected *Pm*-linked locus combination are given in Table S3.

To test whether a greater number of marker-detected *Pm*-linked loci improved powdery mildew resistance, the 660 lines were grouped into 0, 1, 2, 3, and 4 gene classes (Table 7). For E15, the mean infection types were 2.60, 2.66, 2.46, 2.55, and 2.75, respectively, and no significant difference among gene-count classes was detected (Kruskal–Wallis *p* = 0.384; Spearman rho = −0.043, *p* = 0.275). For E17, the mean infection types were 2.45, 2.26, 2.50, 1.96, and 3.25, respectively. The class effect was significant for E17 (Kruskal–Wallis *p* = 0.002), but the monotonic correlation between gene number and infection type was not significant (Spearman’s rho = −0.028, *p* = 0.470). The three-gene class had the highest proportion of lines resistant to E17 (IT ≤ 2; 44/67, 65.7%), whereas the four-gene class had only one line resistant to E15 (1/4, 25.0%) and one line resistant to E17 (1/4, 25.0%). These results do not support a simple model in which more marker-detected *Pm*-linked loci always confer stronger seedling resistance. Powdery mildew resistance was race dependent, and some marker-detected *Pm*-linked locus combinations showed different resistance patterns. Therefore, effective pyramiding should prioritize gene identity and complementarity rather than gene number alone. To further examine whether resistance patterns were associated with particular locus combinations rather than locus number alone, marker-detected *Pm*-linked locus combinations were compared using line-level phenotypic data (Table S3). Because several combinations contained limited numbers of lines, these comparisons were considered exploratory.

### 3.7. Identification of Elite Lines

By combining agronomic performance, powdery mildew seedling response, and stripe rust resistance under natural field disease conditions and marker-detected *Pm*-linked loci, 19 elite wheat lines were identified (Table 8). These lines maintained acceptable plant height (63.09–110.75 cm) and relatively high thousand-grain weight (40.54–56.28 g), indicating that resistance improvement was not accompanied by obvious agronomic penalties. The selected elite lines showed diverse combinations of powdery mildew and stripe rust resistance together with favorable agronomic performance (Table 8). Most lines exhibited resistant or highly resistant responses to both *Bgt* races and stripe rust under field conditions, although the resistance profiles varied among individual lines. Several lines, including SWUST-702, SWUST-716, and SWUST-718, showed strong resistance to both powdery mildew and stripe rust, whereas other elite lines combined favorable disease resistance with different marker-detected *Pm*-linked locus backgrounds. For example, SWUST-675 carried marker-detected *Pm*-linked loci corresponding to *Pm1c*, *Pm52*, and *Pm45* and exhibited strong resistance performance together with acceptable agronomic traits. Overall, these elite lines were selected based on the integration of disease resistance, agronomic performance, and marker-detected *Pm*-linked locus information, rather than resistance performance alone, providing diverse germplasm resources for wheat disease-resistance breeding.

## 4. Discussion

### 4.1. Agronomic Traits

In this study, multienvironment agronomic data, disease resistance phenotyping, and *Pm* marker detection were used to characterize 660 wheat lines derived from resistance-oriented crosses. The cleaned agronomic dataset revealed that TGW, PH, SL, and SN were continuously distributed, indicating that the population retained variation in yield-related traits rather than being restricted to a narrow agronomic background. The shift toward shorter plants in 2024XJ suggests that plant height was more sensitive to the environment than to spikelet number and spike length, whereas the relatively stable distributions of SL and SN indicate that spike architecture was less affected by year or site. The significant associations between SL and both SN and TGW indicate that SL could be used as an auxiliary selection characteristic to improve spike design and grain weight. The mild and environment-dependent SN–TGW trade-off found here is consistent with prior findings, implying that selection for higher spikelet number may not always reduce grain weight. The positive associations of SL with both SN and TGW suggest that longer spikes may contribute to sink capacity and grain weight, but the negative SN–TGW relationship also indicates a compensation effect when the spikelet number increases. Selection in this population may need to balance spike length and grain weight rather than target a single yield component. The moderate heritability observed for SN and SL relative to PH and TGW is consistent with previous reports that spike-architecture traits are generally more sensitive to environmental variation than plant stature [47].

Genotype × environment interaction was substantial in this population. Variance component analysis indicated that the G × E component accounted for 63.9–92.6% of the combined genetic and interaction variance across traits (81.5% for PH, 92.6% for SL, 84.2% for SN, 63.9% for TGW, 76.2% for IT and 76.3% for DS), and cross-environment correlations within traits were correspondingly low to moderate (mean pairwise r = 0.15–0.36). These values confirm that environmental sensitivity is a major determinant of phenotype in this germplasm set, as reported for other multienvironment wheat trials [2,3]. Because each genotype × environment combination was represented by a single observation, the G × E term could not be tested for significance; the interaction mean square was instead used as the error term for testing the genotype and environment main effects.

### 4.2. Disease Resistance Phenotypes

The disease resistance results show that powdery mildew resistance and stripe rust resistance should be evaluated as partly independent breeding targets. The weak association between seedling responses to E15 and E17 indicates clear race specificity and is consistent with the race-specific nature of many seedling resistance genes [4,6,45]. This pattern may reflect the presence of race-specific *Pm*-linked loci, allelic differences among marker-detected loci, or additional background resistance not captured by the markers used in this study. Because only two *Bgt* races were tested, the resistance spectrum of the 22 highly resistant lines remains to be fully defined. Future evaluation using a broader panel of *Bgt* isolates from different wheat-growing regions will be necessary to determine whether these lines provide broad-spectrum or durable resistance [4,78]. The stripe rust response was strongly shaped by the field environment, with 2022QL showing the highest IT and DS values and the strongest IT–DS correlation. Therefore, high-pressure field environments are needed to distinguish partial resistance from near immunity, as emphasized in previous stripe rust studies [41,50]. The high frequency of stripe rust resistance in this population is likely related to its breeding origin, as the lines were derived from crosses involving resistant donors and were subjected to marker-assisted selection and field screening during population development. Nevertheless, the clear environmental differentiation observed in the field trials, particularly the broader IT and DS distributions in 2022QL, confirms that high-pressure environments remain essential for distinguishing partial resistance from near immunity. The weak correlations between *Bgt* infection types and *Pst* traits further suggest that the two disease-resistance systems are controlled by different genetic components; thus, dual resistance likely requires deliberate selection rather than being expected from selection for one disease alone [2,35].

The high level of stripe rust resistance observed in many donor-derived lines is consistent with previous reports that high-level resistance can be recovered and maintained when resistance sources are transferred into adapted backgrounds [35,36]. The performance of SWUST-716, SWUST-718, and SWUST-675 indicates that useful resistance can be recovered in agronomically acceptable backgrounds. However, the documented vulnerability of widely deployed stripe rust resistance sources to new or regional *Pst* populations [79] means that these materials should not be used as single-gene solutions. Their breeding value is in combining field resistance with multienvironment screening and, where possible, additional resistance loci. For powdery mildew, the predominance of moderate and susceptible reactions among a substantial portion of the population is consistent with previous reports indicating that several historically deployed *Pm* genes have lost or are losing effectiveness in China [80]. These results support a combined strategy in which phenotypic screening verifies actual resistance and marker-assisted selection tracks known *Pm* loci.

Earlier evaluations of wheat cultivars and lines reported substantial variation in powdery mildew resistance across *Bgt* populations and gradual loss of effectiveness of several historically used *Pm* genes in China [80]. The present donor-derived breeding population showed a similar race-dependent pattern. In this study, compared with E17, E15 produced a higher mean infection type, and only 22 lines (3.3%) were highly resistant to both races (IT ≤ 1 to both). Therefore, resistance identified with one *Bgt* race should not be regarded as broad-spectrum resistance without additional isolate testing. Marker-based detection should be combined with multirace phenotyping when selecting powdery mildew-resistant wheat germplasms.

### 4.3. Marker Screening

Marker detection revealed that *Pm52*, *Pm42*, *Pm1c*, and *Pm45* were the predominant genes in the population, whereas several target genes, including *Pm21*/*PmV*, were absent from the parental materials and therefore could not contribute to the derived lines. Because *Pm21* and *PmV* were assayed with the same MBH1 marker, the absence of the marker band indicates the absence of the MBH1-tagged haplotype rather than the independent absence of each locus. It should be noted that the molecular markers used in this study detect marker alleles linked to previously reported *Pm* loci. Therefore, marker presence does not necessarily prove the presence, expression, or functionality of the corresponding resistance gene [6,26,78]. False-positive amplification, recombination between markers and causal genes, allelic variation, and genetic-background effects may affect the relationship between marker genotype and disease phenotype. Accordingly, we refer to these loci as marker-detected *Pm*-linked loci rather than functionally confirmed resistance genes throughout this manuscript. This absence is noteworthy, as *Pm21* has been widely regarded as an effective resistance source and is frequently monitored in breeding programs [49,81,82,83]. Consequently, the resistance observed in the present population represents lines without the MBH1-tagged *Pm21*/*PmV* background, which may help diversify the genetic basis of powdery mildew resistance in breeding materials. However, because MBH1 is a marker associated with *Pm21* and *PmV* rather than a direct functional assay, the absence of MBH1 amplification cannot completely exclude the possibility of functional *Pm21* alleles or allelic variants not captured by this marker.

Marker-count analysis indicated that the number of marker-detected *Pm*-linked loci did not show a universal additive relationship with *Bgt* resistance. Although lines carrying three marker-detected *Pm*-linked loci showed improved resistance to race E17 compared with lines carrying fewer such loci, this pattern should be interpreted as an association rather than direct evidence of functional gene pyramiding; however, lines with four genes did not exhibit superior resistance, and no significant trend was observed across gene-count classes for race E15. These findings suggest a possible combination-associated pattern rather than a simple number-dependent effect; however, functional validation and additional phenotyping are required before confirming specific gene-combination effects. Marker-assisted pyramiding may be a useful strategy, but phenotypic validation is also important across multiple *Bgt* isolates rather than by gene count alone, as recent analyses of resistance gene deployment in Chinese wheat cultivars have shown [7,26].

### 4.4. Germplasm Screening

The 19 selected lines represent useful germplasms for dual-disease resistance breeding, as they combine low disease responses with acceptable agronomic performance. Across this set, plant height ranged from 63.09 to 110.75 cm, thousand-grain weight from 40.54 to 56.28 g, spikelet number per spike from 14.08 to 20.04, and spike length from 8.29 to 11.56 cm (Table 8), indicating that the observed resistance was not achieved at the cost of obvious agronomic penalties. Three lines, SWUST-702, SWUST-716 and SWUST-718, showed immune reactions (IT = 0) to both *Bgt* races E15 and E17. Among them, SWUST-716 and SWUST-718 additionally showed highly resistant field responses to stripe rust (IT = 1.75 in both cases; DS = 13.05% and 15.68%, respectively) together with favorable agronomic traits (PH = 87.10 and 90.76 cm; TGW = 49.17 and 50.85 g), suggesting that they may merit consideration as parental candidates; SWUST-702 showed a comparatively less favorable stripe rust response (IT = 2.50; DS = 18.80%). SWUST-675, which combined a low stripe rust response (IT = 1.75; DS = 9.53%) with three marker-detected *Pm*-linked loci (*Pm1c*, *Pm52* and *Pm45*) and acceptable agronomic performance, could also be of interest. It should be emphasized that the stripe rust responses of these lines were highly resistant rather than immune, and that the resistance patterns of SWUST-675, SWUST-716 and SWUST-718 should not be interpreted as confirmed novel resistance-gene combinations on the basis of marker detection alone: SWUST-716 and SWUST-718 carried only one (*Pm52*) and two (*Pm45* and *Pm1c*) marker-detected *Pm*-linked loci, respectively, and the correspondence between the detected marker alleles and functional resistance remains unverified. Rather, these lines represent promising phenotypic sources that combine strong resistance to the tested *Bgt* races with highly resistant stripe rust responses in acceptable agronomic backgrounds. Their novelty lies in the integration of dual-disease resistance and favorable agronomic performance in adapted breeding materials, whereas the underlying resistance genes or allelic variants require further genetic and functional validation [35,38,57]. These lines may contribute to broadening the disease resistance base of adapted wheat backgrounds, particularly when they are crossed with materials carrying additional effective *Pm* or *Yr* loci.

Despite the strong G × E interaction described above, the 19 selected lines showed comparatively consistent resistance across environments. Eleven of the 19 lines (57.9%) maintained IT ≤ 3 in all four environments, compared with 28.8% (190/659) in the full population (Fisher’s exact test, odds ratio = 3.39, *p* = 0.0098), and 89.5% met this threshold in at least three of four environments. Stability was also evident under the most severe disease pressure: in 2022QL, where only 33.1% of the population showed IT ≤ 3, 63.2% of the selected lines remained resistant, and 63.2% maintained DS ≤ 20% (compared with 30.8% population-wide). This combination of multi-environment consistency and performance under high disease pressure supports the use of these lines as parental material, although their stability should be confirmed in additional years and locations [33,36].

Several limitations should be considered. First, powdery mildew resistance was evaluated using only two *Bgt* races; thus, the breadth and durability of the selected lines should be tested against additional isolates from different wheat-growing regions. Second, stripe rust resistance was evaluated across several environments, but additional years and high-pressure locations are needed to confirm its stability. Third, marker detection covered only 14 known *Pm* genes and could not identify unknown resistance loci or allele-level variation. Future work should combine broader pathogen testing, adult-plant powdery mildew evaluation, fine mapping of major stripe rust resistance sources, and genomic analysis of gene combinations. The lines identified in this study integrate useful agronomic traits with resistance to powdery mildew and stripe rust and provide material for pyramiding resistance genes in wheat breeding programs.

### 4.5. Unexplained Resistance, Gene Pyramiding Limits, and Implications for Dual-Resistance Breeding

Notably, 158 lines carrying none of the detected *Pm* markers still exhibited moderate to high resistance to *Bgt* races E15 and E17 (Table 7). For example, 46.8% of these marker-negative lines presented an IT ≤ 2 against E15 and 51.9% against E17, strongly suggesting the presence of unknown or unmarked powdery mildew resistance loci in the population. These putative novel genes may originate from the donor parents or from recombination events during backcrossing [5,78,84]. Future work should employ genome-wide association studies (GWASs) or bulked segregant analysis to map these cryptic resistance sources, which could broaden the genetic basis of powdery mildew resistance beyond currently deployed *Pm* genes [4,23,78]. Bulked segregant analysis coupled with RNA sequencing (BSR-Seq) provides a particularly direct route to such loci: this approach recently delimited *PmL270*, a new powdery mildew resistance gene from wheat line L270, to a 0.1-cM (630-kb) interval on chromosome 7AL that is distinct from all *Pm* genes previously reported on that arm, and yielded the co-dominant marker X7AL08 for marker-assisted introgression [85].

The observation that lines with three marker-detected *Pm*-linked loci showed significantly better resistance to E17 than two-gene lines did but that four-gene lines did not show further improvement challenges the simple additive model of gene pyramiding. Several mechanisms may explain this phenomenon: (i) epistatic interference among coexpressed NLR proteins, where excessive immune receptors compete for downstream signaling components; (ii) race-specificity mismatch of additional genes may not be effective against the tested races; and (iii) expression level thresholds beyond a certain number of resistance genes and no further reduction in infection type are achievable. These findings indicate that marker-assisted pyramiding should prioritize functional complementarity (e.g., combining genes with different recognition spectra) rather than maximizing gene count [6,7,26].

Given the weak correlation between powdery mildew resistance and stripe rust resistance (Figure 3d), this independence has two important breeding implications. First, dual resistance cannot be achieved by selecting for one disease alone; separate screening pipelines for *Bgt* and *Pst* are essential. Second, the lack of a trade-off between the two resistance systems suggests that stacking both traits is feasible without a yield penalty, as demonstrated by the 19 elite lines in Table 8 and consistent with previous dual-resistance breeding efforts [36,60].

Compared with previously reported resistant germplasms, the 19 elite lines identified in this study represent diverse resistance resources combining powdery mildew resistance, stripe rust resistance, and acceptable agronomic performance (Table 8). Among these lines, SWUST-716 and SWUST-718 showed strong resistance responses to both *Bgt* races and stripe rust while maintaining favorable agronomic traits. SWUST-716 exhibited a plant height of 87.10 cm and a thousand-grain weight (TGW) of 49.17 g, whereas SWUST-718 showed a plant height of 90.76 cm and a TGW of 50.85 g. Marker analysis indicated that these lines carried different marker-detected *Pm*-linked loci, but no MBH1-tagged *Pm21*/*PmV* haplotype was detected. Given that *Pm21* has been widely deployed in wheat breeding and faces potential durability challenges under strong selection pressure [86], diversifying resistance resources beyond the MBH1-associated *Pm21*/*PmV* background is valuable for sustainable resistance breeding. Therefore, these materials should be considered valuable resistance resources without the detected MBH1-associated *Pm21*/*PmV* background rather than confirmed *Pm21*-independent resistance sources. In addition, marker-negative resistant lines, such as SWUST-1 and SWUST-135, may contain resistance factors not captured by the current marker panel and represent potential materials for further genetic analysis [38,39,59].

## 5. Conclusions

This study identified wheat germplasm lines that combine favorable agronomic performance with resistance to powdery mildew and stripe rust [2,41]. Multienvironment phenotyping, seedling-stage powdery mildew screening, stripe rust evaluation under natural field disease conditions, and marker detection together supported the selection of resistant parental materials and the pyramiding of effective resistance genes in wheat breeding programs [6,7,26]. Gene-count analysis revealed that powdery mildew resistance was not determined simply by the number of marker-detected *Pm*-linked loci [7,49]. Resistance was race dependent and combination specific, with three-gene classes showing improved E17 resistance but four-gene classes not showing increased resistance [4,45,80]. The absence of MBH1 amplification indicates that these lines do not carry the MBH1-tagged *Pm21*/*PmV* haplotype that is commonly monitored in breeding programs [49,66]; however, because MBH1 is a linked rather than a diagnostic marker [66], the presence of functional *Pm21* or *PmV* alleles cannot be fully excluded [49,81]. These lines should therefore be regarded as resistance resources lacking the MBH1-tagged *Pm21*/*PmV* background rather than as confirmed *Pm21*-independent sources [23,78].

## Figures and Tables

**Table 1:** Resistance information of the recipient parents (maternal parents) of the 660 wheat lines.

Name	Source	Stripe Rust Resistance	Powdery Mildew Resistance
Chuanmai 42	Sichuan Academy of Agricultural Sciences	Highly resistant–immune	Moderately susceptible
Bainong Aikang 58	Henan Institute of Science and Technology	Highly resistant	Highly resistant
Han 6172	Handan Academy of Agricultural Sciences, Hebei Province	Highly resistant	Susceptible
Zhengmai 9023	Henan Academy of Agricultural Sciences; Northwest A&F University	Moderately resistant	Highly susceptible
Lunxuan 987	Chinese Academy of Agricultural Sciences	Moderately susceptible	Moderately resistant
Jimai 22	Shandong Academy of Agricultural Sciences	Moderately resistant	Susceptible
Xinmai 26	Xinxiang Academy of Agricultural Sciences, Henan Province; Henan Dunhuang Seed Industry Xinke Seed Co., Ltd.	Moderately susceptible	Highly susceptible
Xiangmai 25	Xiangfan Academy of Agricultural Sciences, Hubei Province	Moderately susceptible	Moderately susceptible
Yannong 21	Yantai Academy of Agricultural Sciences, Shandong Province	Moderately resistant	Moderately resistant

The disease resistance data were retrieved from the China Seed Industry Big Data Platform (http://202.127.42.145/bigdataNew/home/firmindex, accessed on 15 June 2026), which provides the resistance evaluation results for stripe rust and powdery mildew obtained during the official variety registration process.

**Table 2:** *Yr* genes carried by the donor parents (male parents) of the 660 wheat lines.

Name	Carried Stripe Rust Resistance Genes	Reference
JPR02 (PI 660057)	*Yr52*	[51]
JPR05 (PI 660060)	*Yr62*	[52]
JPR06 (PI 660061)	*Yr59*	[53]
JPR21 (PI 660076)	*QYr076.jaas-2A*, *QYr076.jaas-4D.1*, *QYr076.jaas-4D.2*	[54]
JPR60 (PI 660115)	Unknown *	\
JPR67 (PI 660122)	*QYrPI660122.swust-4BS*, *QYrPI660122.swust-4BL*, *QYrPI660122.swust-4DS*, *QYrPI660122.swust-4DL*, *QYrPI660122.swust-7DS*	[55]
JPR75 (PI 610750)	*Yr48*	[56]
JPR77 (AvS/Express F_7_)	*YrExp2*	[57]
JPR80 (AvS/Alp F_7_-71)	*Yr39*	[58]
P9897	*QYr.nafu-2BL*, *QYr.nafu-3BS*	[60]

* The stripe rust resistance gene in JPR60 (PI 660115) has not been formally identified.

**Table 3:** Markers and their primers for detecting powdery mildew resistance genes.

Gene	Marker	Primer Sequence	Reference
*Pm1c*	*Xbarc78-F*	CTCCCCGGTCAAGTTTAATCTCT	[61]
	*Xbarc78-R*	GCGACATGGGAATTTCAGAAGTGCCTAA	
*Pm2*	*CFD81-F*	TATCCCCAATCCCCTCTTTC	[62]
	*CFD81-R*	GTCAATTGTGGCTTGTCCCT	
*Pm4a*	*Xgwm356-F*	AGCGTTCTTGGGAATTAGAGA	[63]
	*Xgwm356-R*	CCAATCAGCCTGCAACAAC	
*Pm5e*	*WMC364-F*	ATCACAATGCTGGCCCTAAAAC	[64]
	*WMC364-R*	CAGTGCCAAAATGTCGAAAGTC	
*Pm6*	*CIT02g-18-F*	GGCCTTAGTGGTGATGCAGT	[65]
	*CIT02g-18-R*	GCGGCTTGTCGGTGTATAG	
*Pm21/PmV*	*MBH1-F*	GCCATTATAGTCAAGAGTGCACTAGCTGT	[66]
	*MBH1-R*	AGCTCCTCTCGTTCTCCAATGCT	
*Pm24*	*STS-Pm24-F*	TATGGTGTCATTTAAGGCTGAG	[67]
	*STS-Pm24-R*	TTTCTCACATCCTCATCAAACC	
*Pm30*	*Xgwm159-F*	GGGCCAACACTGGAACAC	[68]
	*Xgwm159-R*	GCAGAAGCTTGTTGGTAGGC	
*Pm33*	*GWM111-F*	TCTGTAGGCTCTCTCCGACTG	[64]
	*GWM111-R*	ACCTGATCAGATCCCACTCG	
*Pm35*	*CFD26-F*	TCAAGATCGTGCCAAATCAA	[69]
	*CFD26-R*	ACTCCAAGCTGAGCACGTTT	
*Pm42*	*Xgwm148-F*	GTGAGGCAGCAAGAGAGAAA	[69]
	*Xgwm148-R*	CAAAGCTTGACTCAGACCAAA	
*Pm45*	*CFD80-F*	ATAGGGGTTTTGAATCACTCC	[63]
	*CFD80-R*	TTGGATTTGCAGAGCCTTCT	
*Pm52*	*Xicscl795-F*	GTCAACCTCATCTTCTCCTG	[70]
	*Xicscl795-R*	AGATGCATATCACATTCACG	
*Pm68*	*Xdw15-F*	GCTAATTACTACTCTCTTCGTTCCGA	[71]
	*Xdw15-R*	GAATATGACCCAACAAATATCCGACA	

*Pm21* and *PmV* were detected with the same MBH1 marker because the two loci share this marker; they are therefore treated as a single marker-detected unit (*Pm21*/*PmV*) rather than two independent loci. Fourteen marker pairs corresponding to 14 known *Pm* loci were used in this study.

**Table 4:** Two-way analysis of variance for agronomic and disease resistance traits.

Trait	Genotype	Environment	G × E	H^2^ (%)
PH	4.86 ***	253.93 ***	56.72	79.42
SL	3.03 ***	29.29 ***	1.17	66.95
SN	2.58 ***	3.85 **	2.03	61.23
TGW	3.35 ***	52.69 ***	22.58	70.14
IT	1.88 ***	538.34 ***	1.01	46.69
DS	2.10 ***	646.42 ***	75.51	52.30

Values for genotype and environment are F values from the two-way ANOVA, whereas the genotype-by-environment interaction is given as a mean square (MS). Because each genotype × environment combination was represented by a single observation, the residual and interaction terms were confounded; the G × E mean square was therefore used as the error term for testing the genotype and environment main effects, and the G × E term itself could not be tested for significance. H^2^ indicates broad-sense heritability. ** and *** indicate significance at *p* < 0.01 and *p* < 0.001, respectively. Abbreviations: PH, plant height; SL, spike length; SN, spikelet number; TGW, thousand-grain weight; IT, infection type; DS, disease severity.

**Table 5:** Descriptive statistics and ANOVA information by environment.

Trait	Environment	Min	Max	Mean	CV (%)	Ske	Kur	Genotype	Env_F	G×E	H^2^ (%)
PH	2019QL	72.00	125.00	97.23	10.26	0.274	−0.063	4.86 ***	253.93 ***	56.72	79.42
PH	2020QL	70.00	123.00	96.17	10.52	0.265	−0.161	4.86 ***	253.93 ***	56.72	79.42
PH	2020YL	75.00	121.40	98.28	8.64	0.157	−0.079	4.86 ***	253.93 ***	56.72	79.42
PH	2022QL	62.00	130.00	95.43	12.82	0.199	−0.333	4.86 ***	253.93 ***	56.72	79.42
PH	2024XJ	60.55	111.50	86.43	10.84	0.231	−0.053	4.86 ***	253.93 ***	56.72	79.42
SL	2019QL	7.20	12.60	9.89	10.45	0.103	−0.328	3.03 ***	29.29 ***	1.17	66.95
SL	2020QL	6.00	13.00	9.67	14.78	−0.000	−0.025	3.03 ***	29.29 ***	1.17	66.95
SL	2020YL	6.50	14.00	10.23	13.95	0.022	−0.421	3.03 ***	29.29 ***	1.17	66.95
SL	2022QL	7.00	12.67	9.70	11.84	0.191	−0.338	3.03 ***	29.29 ***	1.17	66.95
SL	2024XJ	6.25	13.70	10.01	13.91	0.269	−0.412	3.03 ***	29.29 ***	1.17	66.95
SN	2020QL	14.00	22.33	18.00	10.11	0.049	−0.504	2.58 ***	3.85 **	2.03	61.23
SN	2020YL	14.00	22.00	17.86	9.26	0.057	−0.358	2.58 ***	3.85 **	2.03	61.23
SN	2022QL	13.33	22.33	17.79	8.86	0.061	−0.093	2.58 ***	3.85 **	2.03	61.23
SN	2024XJ	13.50	23.00	18.02	9.75	−0.090	−0.331	2.58 ***	3.85 **	2.03	61.23
TGW	2019QL	30.40	61.20	45.74	12.86	0.176	−0.189	3.35 ***	52.69 ***	22.58	70.14
TGW	2020QL	30.80	66.70	48.28	13.18	0.064	−0.238	3.35 ***	52.69 ***	22.58	70.14
TGW	2022QL	29.59	60.90	45.22	12.54	−0.176	−0.241	3.35 ***	52.69 ***	22.58	70.14
TGW	2024XJ	30.82	62.27	46.89	13.34	−0.072	−0.264	3.35 ***	52.69 ***	22.58	70.14
IT	2019QL	0.80	4.00	2.45	27.94	0.314	−0.011	1.88 ***	538.34 ***	1.01	46.69
IT	2020QL	1.00	4.00	2.36	25.73	0.861	0.479	1.88 ***	538.34 ***	1.01	46.69
IT	2020YL	1.00	4.00	2.62	28.76	0.205	−0.503	1.88 ***	538.34 ***	1.01	46.69
IT	2022QL	0.00	8.67	4.30	44.12	0.125	−0.947	1.88 ***	538.34 ***	1.01	46.69
DS	2019QL	0.00	40.00	13.86	73.26	0.954	0.220	2.10 ***	646.42 ***	75.51	52.30
DS	2020QL	0.00	20.00	7.36	77.91	0.841	−0.255	2.10 ***	646.42 ***	75.51	52.30
DS	2020YL	0.00	40.00	13.54	69.08	0.651	−0.212	2.10 ***	646.42 ***	75.51	52.30
DS	2022QL	0.00	61.67	27.99	44.70	0.026	−0.642	2.10 ***	646.42 ***	75.51	52.30
E15	2024XJ	1.00	4.00	2.61	34.34	−0.051	−0.781				
E17	2024XJ	1.00	4.00	2.43	38.63	0.075	−0.881				
E15 vs. E17	Mann–Whitney U test							U = 227,305.00	*p* = 0.000530	***	

** and *** indicate significance at *p* < 0.01 and *p* < 0.001, respectively. Abbreviations: PH, plant height; SL, spike length; SN, spikelet number; TGW, thousand-grain weight; IT, infection type; DS, disease severity; CV, coefficient of variation. Comparison between *Bgt* races E15 and E17.

**Table 6:** Distribution of detected powdery mildew resistance genes among parental wheat lines.

Variety	CM42	YN21	H6172	XM26	JM22	ZM9023	Ak58	XM25	LX987	JPR02	JPR05	JPR06	JPR58	JPR67	JPR75	JPR77	JPR80	P9897
*Pm1c*	−	−	−	+	−	+	−	−	−	−	−	−	−	−	−	−	−	−
*Pm2*	−	−	−	−	+	−	−	−	+	−	−	−	−	−	−	−	−	−
*Pm4a*	−	−	−	−	−	−	−	−	−	−	−	−	−	−	−	−	−	−
*Pm5e*	−	−	−	−	−	−	−	−	−	+	+	−	−	−	−	−	−	−
*Pm6*	−	−	−	−	+	−	−	−	+	−	−	−	−	−	−	−	−	−
*Pm21*	−	−	−	−	−	−	−	−	−	−	−	−	−	−	−	−	−	−
*Pm24*	−	−	−	−	−	−	−	−	−	−	−	−	−	−	−	−	−	−
*Pm30*	−	−	−	−	−	−	+	+	−	−	+	−	−	−	−	−	−	−
*Pm33*	−	−	−	−	−	−	−	−	−	−	−	−	−	−	−	−	−	−
*Pm35*	−	−	−	−	−	−	−	−	−	−	−	−	−	−	−	−	−	−
*Pm42*	−	−	−	−	−	−	+	−	−	+	+	+	+	−	−	−	+	−
*Pm45*	−	−	−	+	−	+	+	+	−	−	+	−	−	−	−	−	−	−
*Pm52*	+	−	−	−	−	−	−	+	−	+	−	−	+	+	−	−	−	+
*Pm68*	−	−	−	−	−	−	−	−	−	−	−	−	−	−	−	−	−	−
*PmV*	−	−	−	−	−	−	−	−	−	−	−	−	−	−	−	−	−	−

+ indicates that the parental line carries the corresponding powdery mildew resistance gene; − indicates the absence of the gene.

**Table 7:** Powdery mildew responses grouped by the number of marker-detected *Pm*-linked loci.

Marker-Detected *Pm*-Linked Loci	Lines (n)	E15 Valid n	E15 Mean IT ± SE	E15 IT ≤ 1 n (%)	E15 IT ≤ 2 n (%)	E17 Valid n	E17 Mean IT ± SE	E17 IT ≤ 1 n (%)	E17 IT ≤ 2 n (%)
0	158	156	2.60 ± 0.08	18 (11.5%)	73 (46.8%)	156	2.45 ± 0.07	22 (14.1%)	81 (51.9%)
1	240	235	2.66 ± 0.06	22 (9.4%)	93 (39.6%)	236	2.26 ± 0.07	54 (22.9%)	137 (58.1%)
2	189	178	2.46 ± 0.08	32 (18.0%)	88 (49.4%)	187	2.50 ± 0.08	36 (19.3%)	91 (48.7%)
3	69	66	2.55 ± 0.13	10 (15.2%)	33 (50.0%)	67	1.96 ± 0.15	23 (34.3%)	44 (65.7%)
4	4	4	2.75 ± 0.25	0 (0.0%)	1 (25.0%)	4	3.25 ± 0.48	0 (0.0%)	1 (25.0%)

An IT ≤ 1 indicates immune or highly resistant responses; an IT ≤ 2 indicates resistant responses, including moderately resistant lines. A lower infection type indicates stronger resistance. Missing phenotype values were excluded from the corresponding valid n. SE, standard error.

**Table 8:** Agronomic traits, disease-resistance phenotypes, and marker-detected *Pm*-linked loci in the selected wheat lines.

Line	PH (cm)	TGW (g)	SN	SL (cm)	IT (Pst)	DS (Pst)	IT (E15)	IT (E17)	Marker-Detected *Pm*-Linked Loci
SWUST-1	92.54 ± 3.06	41.78 ± 3.61	16.79 ± 0.98	8.29 ± 0.23	2.85	18.07	1	1	None
SWUST-135	63.09 ± 0.87	41.32 ± 4.76	20.04 ± 0.50	10.08 ± 0.51	2.67	17.08	1	1	None
SWUST-28	95.50 ± 4.93	49.46 ± 1.66	16.67 ± 0.94	9.72 ± 0.46	2.50	16.67	1	1	*Pm30*
SWUST-306	103.41 ± 3.41	44.22 ± 1.32	18.08 ± 0.48	9.10 ± 0.35	2.08	9.17	1	1	*Pm52*
SWUST-349	110.75 ± 6.02	56.28 ± 2.10	19.00 ± 0.83	10.04 ± 1.45	2.26	7.21	1	1	*Pm42*
SWUST-612	89.79 ± 4.54	41.84 ± 4.19	17.45 ± 0.57	9.91 ± 0.47	2.50	8.14	1	0	*Pm45*
SWUST-625	94.16 ± 6.70	48.29 ± 1.34	19.16 ± 0.82	9.72 ± 0.40	1.75	8.72	1	1	*Pm1c*
SWUST-706	103.60 ± 5.04	54.79 ± 3.93	14.19 ± 2.16	9.77 ± 0.62	2.25	16.04	1	1	*Pm52*
SWUST-707	107.20 ± 4.29	54.13 ± 3.22	14.08 ± 1.81	10.27 ± 0.60	2.00	12.40	1	1	*Pm52*
SWUST-716	87.10 ± 1.62	49.17 ± 3.52	16.68 ± 1.23	9.77 ± 0.77	1.75	13.05	0	0	*Pm52*
SWUST-750	92.81 ± 5.40	47.60 ± 1.93	17.82 ± 0.63	9.15 ± 1.00	2.75	17.39	0	1	*Pm1c*
SWUST-504	85.04 ± 6.19	47.65 ± 3.97	18.25 ± 1.03	11.41 ± 0.76	2.94	19.83	1	1	*Pm52*, *Pm42*
SWUST-717	91.01 ± 1.80	53.72 ± 2.62	15.13 ± 0.86	10.35 ± 0.57	2.79	19.23	0	1	*Pm45*, *Pm1c*
SWUST-718	90.76 ± 6.92	50.85 ± 0.85	16.51 ± 1.47	9.98 ± 0.56	1.75	15.68	0	0	*Pm45*, *Pm1c*
SWUST-26	106.48 ± 7.08	43.32 ± 1.26	17.08 ± 0.34	9.21 ± 0.12	2.98	19.72	1	1	*Pm30*, *Pm52*, *Pm42*
SWUST-502	85.42 ± 2.87	44.95 ± 2.31	18.42 ± 0.25	10.78 ± 0.67	2.86	14.25	1	0	*Pm52*, *Pm42*, *Pm45*
SWUST-675	76.03 ± 11.31	40.54 ± 1.82	17.81 ± 0.82	11.56 ± 0.51	1.75	9.53	1	1	*Pm1c*, *Pm52*, *Pm45*
SWUST-693	95.94 ± 3.38	46.82 ± 0.92	17.68 ± 1.00	10.16 ± 0.48	2.17	12.52	1	0	*Pm1c*, *Pm52*, *Pm45*
SWUST-702	89.71 ± 4.45	51.39 ± 2.39	14.72 ± 2.68	9.88 ± 0.29	2.50	18.80	0	0	*Pm1c*, *Pm52*, *Pm45*

PH, plant height; TGW, thousand-grain weight; SN, spikelet number per spike; SL, spike length. IT (*Pst*) and DS (*Pst*) represent the infection type and disease severity of stripe rust, respectively, assessed under natural field disease conditions. IT (E15) and IT (E17) represent infection types after inoculation with *Bgt* races E15 and E17, respectively. Agronomic traits were evaluated in 2024XJ; stripe rust responses were evaluated in 2019QL, 2020QL, 2020YL and 2022QL. Values are means of three replicate measurements from randomly selected plants and are presented as mean ± SE. Marker-detected *Pm*-linked loci were detected with the markers listed in Table 3.

## Data Availability

The data generated or analysed during this study are available from the corresponding authors upon reasonable request. Germplasm materials are available from the corresponding author upon reasonable request and subject to institutional regulations.

## Supplementary Materials

The following supporting information can be downloaded at: https://www.mdpi.com/article/10.3390/plants15182859/s1. Table S1: Pedigree information of the 660 wheat lines; Table S2: Agronomic traits and disease resistance of the 660 wheat lines; Table S3: Number of lines and *Bgt* responses for each marker-detected *Pm*-linked locus combination.
